# An Intelligent System for Pigeon Egg Management: Integrating a Novel Lightweight YOLO Model and Multi-Frame Fusion for Robust Detection and Positioning

**DOI:** 10.3390/s25237132

**Published:** 2025-11-21

**Authors:** Yufan Cheng, Yao Liu, Qianhui Li, Tao Jiang, Chengyue Ji, Longshen Liu, Ya Zhong, Jinling Wu, Guanchi Chen

**Affiliations:** 1 College of Smart Agriculture (College of Artificial Intelligence), Nanjing Agricultural University, Nanjing 211800, China; 9233020730@stu.njau.edu.cn (Y.C.); liulongshen@njau.edu.cn (L.L.);; 2 School of Mechanical and Electrical Engineering, Beijing Institute of Technology, Beijing 100081, China

**Keywords:** pigeon egg recognition, pigeon egg localization, target detection, image processing

## Abstract

To address the issues of high breakage rates and substantial labor costs in pigeon egg farming, this study proposes an intelligent pigeon egg recognition and positioning system based on an improved YOLOv12n object detection algorithm and OpenCV barcode recognition technology. Visual sensors installed on feeding machines were used to collect real-time video data of pigeon cages, with images obtained through frame extraction. The images were annotated using LabelImg to construct a pigeon egg detection dataset containing 1500 training images, 215 validation images, and 215 test images. After data augmentation, the dataset was used to train the pigeon egg recognition model. Additionally, customized barcodes were designed according to actual farm conditions and recognized using OpenCV through preprocessing steps including grayscale conversion, filtering, and binarization to extract positional information. Experimental results demonstrate that the proposed YOLOv12n-pg recognition model requires only 4.9 GFLOPS computational load, contains 1.56 M parameters, and has a model size of 3.5 MB, significantly lower than other models in the YOLO-n series. In inference tests, it achieved 99.4% mAP50 and 83.6% mAP50-95. The implementation of a majority voting method in practical testing further reduced the missed detection rate. The system successfully records “cage location—egg count” information as key-value pairs in a database. This system effectively enables automated management of pigeon eggs, improves recognition performance, and demonstrates higher efficiency and accuracy compared to manual operations, thereby establishing a foundation for subsequent research in pigeon egg recognition.

## 1. Introduction

With the vigorous rise of the pigeon breeding industry in recent years, pigeons have firmly occupied an essential position as the fourth largest poultry breed after chickens, ducks and geese [1]. The egg-laying pigeon breeding industry in China has also shown a strong growth momentum. Pigeon eggs, with their unique nutritional value—rich in protein, iron, calcium, and other nutrients [2], and containing transparent protein that is easy to digest and absorb—have become a high-quality nutritional supplement [3]. However, in the process of breeding egg-laying pigeons, managing pigeon eggs faces many thorny problems, highlighting the key necessity of this research. Compared with the breeding eggs of standard poultry such as chickens, ducks and geese, pigeon eggs have distinct characteristics. Firstly, the average weight of pigeon eggs is only 15 to 30 g, and their volume is significantly smaller than that of conventional poultry eggs. In the breeding environment, manual egg inspection has a high rate of missed detection and low recognition efficiency. Secondly, the thickness of pigeon eggshells is less than 0.5 mm, and their mechanical strength is far lower than that of chicken eggs or duck eggs. In daily breeding, pigeon eggs are very likely to be damaged by the accidental stepping of the parent pigeons in the egg nest. According to the on-site investigation of a standardised breeding farm, two workers are required for daily egg inspection, which takes 2 to 3 h. What is even more serious is that the rate of pigeon egg damage caused by improper manual operation and missed inspection has long remained at 3% to 5%, resulting in direct economic losses of tens of thousands of yuan every year. Therefore, there is an urgent need for an intelligent system that can achieve precise positioning and efficient management of pigeon eggs, in order to fill the technical gap in the field of pigeon egg management and enhance the overall benefits of breeding egg-laying pigeons.

In the development process of poultry egg detection technology, machine vision technology has achieved relatively mature application results in detecting egg products from poultry such as chickens, ducks, and geese. In 2014, the egg automatic grading system proposed by Xu et al. [4] achieved an accuracy rate of over 95% in both positioning and recognition. In 2017, Sunardi et al. [5] completed the recognition of poultry eggs with the help of smartphones, thermal imaging cameras and MATLAB, achieving an accuracy rate of 100%. In 2021, Behaine et al. [6] proposed a digital visual counting model based on the HSV colour space, which improved by 9% compared to the suboptimal model in a highly diverse environment.

Although object detection algorithms based on deep learning (such as the YOLO series [7,8]) have become mature in the field of agricultural visual inspection, especially in the automatic recognition and counting of large-sized poultry eggs like chickens and ducks [4,9], they are still limited by the unique physical properties of pigeon eggs and the complex environment of closed pigeon cages. There are significant obstacles to the direct transfer and application of the above-mentioned technologies.

In terms of model efficiency and deployment, pigeon eggs are small in size and light in weight, making them a typical problem for small target detection. Although the existing general detection models (such as YOLOv7 [7]) perform well on public datasets, their multi-scale detection heads and complex network structures are designed to deal with a wider range of target scales, resulting in a large number of model parameters and high computational costs, making it difficult to ensure real-time performance on embedded platforms. In terms of interference response, pigeon egg detection is confronted with far more complex dynamic scenarios than chicken and duck egg detection. On the one hand, the metal mesh of the pigeon cage will form a structural occlusion in the image; On the other hand, the frequent activities of the parent pigeons (such as returning to the nest and turning the eggs) can cause temporary obstruction to the pigeon eggs and lead to slight rolling of the egg positions. These factors significantly increase the missed detection rate of detection models designed in relatively static environments [5,10] and traditional image processing methods (such as those based on HSV colour space [6,11,12] or Canny edge detection [13,14]). Existing studies have not yet provided effective solutions on how to maintain detection stability under such persistent dynamic interferences. Finally, at the level of system functionality and management granularity, current research mostly focuses on the single task of “counting”. Whether it is the early automatic grading system [4], or the methods based on morphological operations [10] or robot collection [15,16,17], the core objective is to enhance the accuracy and efficiency of counting. However, in modern refined breeding of breeding pigeons, the existing technology lacks functional modules that can automatically associate visual inspection results with physical location information (such as cage position numbers), and thus cannot form “position-quantity” key-value pairs, which restricts the realization of deep management requirements such as egg-laying pattern analysis based on individual nest positions and performance measurement of breeding pigeons.

Based on on-site investigations of large-scale pigeon farms, an automated egg inspection system needs to meet the following key process requirements. First, in terms of recognition accuracy, the system’s missed detection rate should be less than 1%, and the false detection rate should be less than 2%. This strict standard is the core prerequisite for ensuring the accuracy of pigeon egg asset statistics, avoiding distortion of breeding records and direct economic losses due to missed inspections. Secondly, in terms of processing efficiency, the average processing time for a single pigeon position should not exceed 30 s, to match the operation rhythm of the existing feeding machine and be integrated into the daily management process without changing the existing production rhythm. In addition, the system must have a high degree of reliability and be capable of maintaining stable operation in complex breeding site environments where common dynamic interferences such as temporary obstruction by female pigeons and interference from cage and net structures exist. The system design objective of this study is precisely formulated based on the aforementioned clear industrialisation requirements.

To this end, this study is dedicated to systematically addressing the key technical challenges in the automated management of pigeon eggs. By constructing a complete intelligent detection and positioning system, the following three levels of research goals are achieved: At the model architecture level, the aim is to develop a lightweight deep learning model specifically for pigeon egg recognition. Through a series of optimisation strategies such as trimming redundant large and medium-scale detection heads in the YOLOv12n network, introducing the SimAm parameterless attention mechanism, and the WIoU-v3 loss function, the computational load of the model will be controlled while maintaining high average accuracy. Significantly enhance the inference efficiency on embedded devices. At the anti-interference strategy level, efforts are made to build a dynamic detection mechanism based on time redundancy. By designing a fusion algorithm of multi-frame image acquisition and majority voting, it effectively addresses the interference factors such as instantaneous occlusion and micro-movement of egg positions caused by the activities of parent pigeons in the breeding environment, reducing the missed detection rate of the system in complex scenarios. At the system integration level, efforts are made to establish a collaborative working system for visual inspection and barcode positioning. By optimising image preprocessing, contour detection and decoding algorithms, stable barcode recognition is achieved. The position information obtained from decoding is automatically associated with the number of pigeon eggs detected visually, ultimately forming a complete “position-quantity” key-value pair database. Provide a reliable technical solution for the digital management of egg-laying pigeon breeding.

## 2. Materials and Methods

### 2.1. Data Collection and Processing for the Pigeon Egg Recognition Model

#### 2.1.1. Image Data Acquisition

**Figure 1:**
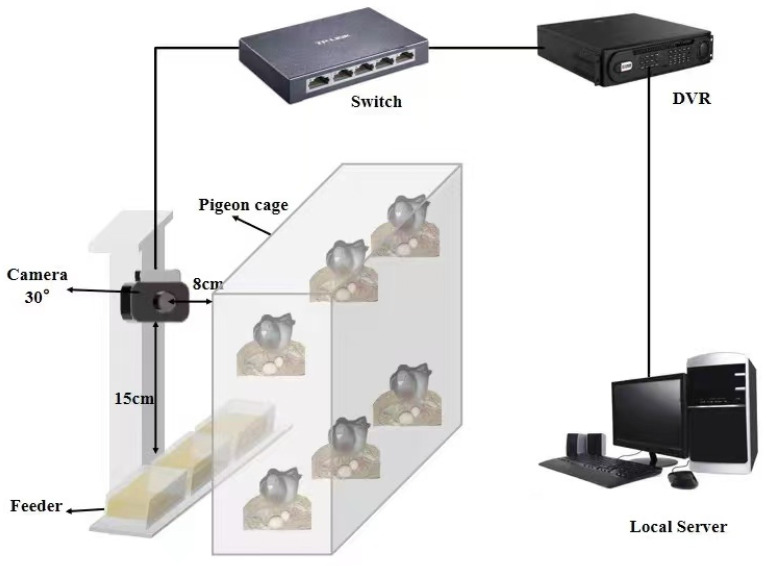
Image data acquisition hardware platform.

The experimental data of this study were sourced from Nanjing Dongchen Pigeon Industry Co., Ltd. in Tuanjie Community, Longpao Sub-district, Liuhe District, Nanjing City, China. From 1 June to 15 June 2024, video data of 150 pairs of breeding pigeon eggs in the factory area of this unit were collected every day, and the Silver King breeding pigeon was selected as the research object. A custom bracket equipped with a Hikvision T12HV3-IA/PoE camera (Hangzhou, China) was installed on the feeding machine of each row of pigeon cages to capture the egg-laying situation inside each cage from the side. The video frames collected are RGB images with a resolution of 1920 × 1080 pixels. The operating speed of the camera is approximately the same as that of the feed machine. Each pigeon cage is 50 cm wide. In each cycle, 50 pairs of egg nests of breeding pigeons are collected, and each collection cycle lasts for 30 min. The collection schematic diagram is shown in Figure 1.

After manual selection to remove unnecessary data, a total video duration of 2 h and 18 min was obtained. The resulting video images underwent frame extraction, producing one image file in .png format every 10 frames. A total of 1930 original photos were obtained.

#### 2.1.2. Image Data Processing

**Figure 2:**
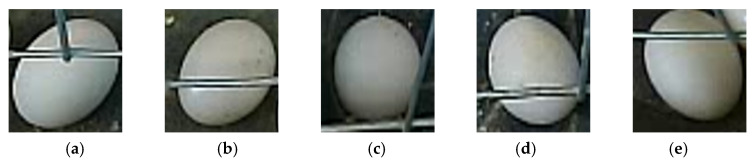
Representative pigeon egg images from the dataset, illustrating varying degrees and types of occlusion caused by metal cage bars (cage nets), which provide crucial data diversity for model training. From left to right: (**a**) rod near the top, (**b**) rod through the middle, (**c**) rod on the side, (**d**) rod near the bottom, (**e**) rod above with a gap.

In this study, pigeon eggs were identified and targeted from the collected image data. Some of the intercepted pigeon eggs are shown in Figure 2. For detecting pigeon egg targets, the LabelImg image annotation tool was used to create the COCO dataset. One thousand nine hundred thirty image data were manually annotated, and the annotation boxes were the smallest rectangular boxes tangent to the periphery of the pigeon eggs. The sizes of the training dataset, validation dataset and test dataset of the model are 1500, 215 and 215, respectively.

#### 2.1.3. The Construction of a Deep Learning Platform

This study trained the model for pigeon egg target detection based on the PyTorch framework [18], and the software and hardware parameters adopted are shown in Table 1 below.

### 2.2. Intelligent Egg-Checking Model Design

#### 2.2.1. Model Network Structure

The object detection networks provided by the YOLOv12 series encompass a spectrum of models, including YOLOv12x, YOLOv12l, YOLOv12m, YOLOv12s, and YOLOv12n [19], which are primarily distinguished by their network depth and width. Among them, YOLOv12n represents the most lightweight and high-speed architecture. The decision to select YOLOv12n as the baseline was driven by the core need to make a fundamental trade-off between accuracy, efficiency, and model size.

To substantiate this choice, preliminary comparisons were conducted on a subset of the dataset between several YOLOv12 family models (e.g., YOLOv12n, YOLOv12s, and YOLOv12m). The results indicated that while the larger models (s, m) offered marginally higher initial accuracy for the specific task, the performance gap was not substantial. Crucially, YOLOv12n demonstrated a decisive advantage in inference speed and model compactness. It was hypothesised that for the highly concentrated scale of pigeon eggs—a typical small object detection problem—the architectural complexity of larger models was unwarranted.

In terms of network structure, YOLOv12 introduces a regional attention module. This design eliminates the window division for display, reduces computational costs, and ensures high efficiency. Furthermore, the adopted R-ELAN structure effectively resolves gradient blocking through an efficient aggregation method, enabling the network to perform better in complex scenarios.

Although YOLOv12 has brought about significant performance improvements and efficiency optimisations. However, it still has some problems in real-time detection of small targets: low accuracy, easy-to-miss detections, and relatively complex calculations. Therefore, this paper innovatively retains only the detection head of YOLOv12 for small targets, while introducing the SimAm attention-free mechanism. This can effectively reduce the model size, lower the computational complexity, and improve the inference accuracy.

#### 2.2.2. Design of Detection Head

The original YOLOv12n adopts multi-scale detection heads (P3, P4, P5) to accommodate targets of different sizes. However, we analysed the scale distribution of pigeon eggs in the dataset and found that they were highly concentrated in the small target range (area < 32^2^ pixels). Therefore, the design of YOLOv12n introduced unnecessary structural redundancy and computational overhead.

**Figure 3:**
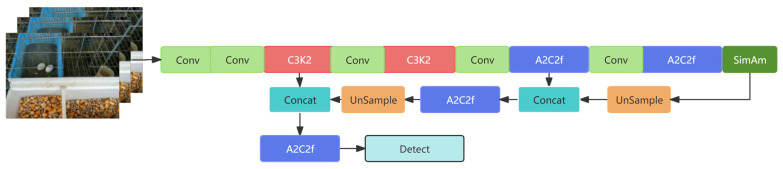
The architecture of the proposed YOLOv12n-pg model. The key modification is the removal of the P4 and P5 detection heads responsible for medium and large targets, retaining only the P3 head optimized for small target (pigeon egg) detection.

To solve this problem, we propose a detection head trimming strategy for pigeon egg recognition. We redesigned the network structure by trimming the P4 and P5 detection heads responsible for medium and large targets and retaining only the P3 detection head, which is most suitable for small targets. This approach constructs a more compact model, as shown in Figure 3.

#### 2.2.3. SimAm’s Parameter-Free Attention Mechanism

In the task of pigeon egg detection, there are numerous interferences due to colour and texture similarities between the background (such as feed, pigeon nests) and the detection target (pigeon eggs). Additionally, the pigeon egg target is relatively small and can be easily blocked by the pigeon’s body. Therefore, the model needs to incorporate an attention mechanism for optimisation. However, classic channel or spatial attention modules (such as SE, CBAM) typically generate weights by introducing fully connected layers or convolutional layers, which inevitably increases the number of parameters and computational complexity of the model, raises the risk of overfitting, and to some extent limits the application of the model in lightweight deployment scenarios.

To solve the above problems, this study introduces A Simple Parameter-Free Attention Module (SimAm) [20]. The classic energy function inspires this module in neuroscience and defines the following energy function for each neuron:

(1) $$e_{t}(w_{t},b_{t},y,x_{i})={\left(y_{t}-\hat{t}\right)}^{2}+\frac{1}{M-1}\sum_{i=1}^{M-1}{\left(y_{o}-{\hat{x}}_{i}\right)}^{2}$$

Among them, $\hat{t}=w_{t}t+b_{t}$ and ${\hat{x}}_{i}=w_{t}x_{i}+b_{t}$ is a linear transformation. Here, t and $x_{i}$ are the target neuron and other neurons in a single channel of the feature map $X\in R^{C\times H\times W}$. M represents the total number of neurons in that channel (i.e., M = H × W), and i is the index over these neurons. $w_{t}$ and $b_{t}$ is the weight and bias of the linear transformation. By minimising this energy function, the importance weight (i.e., the attention value) of each neuron can be calculated without any explicit network layer.

**Figure 4:**
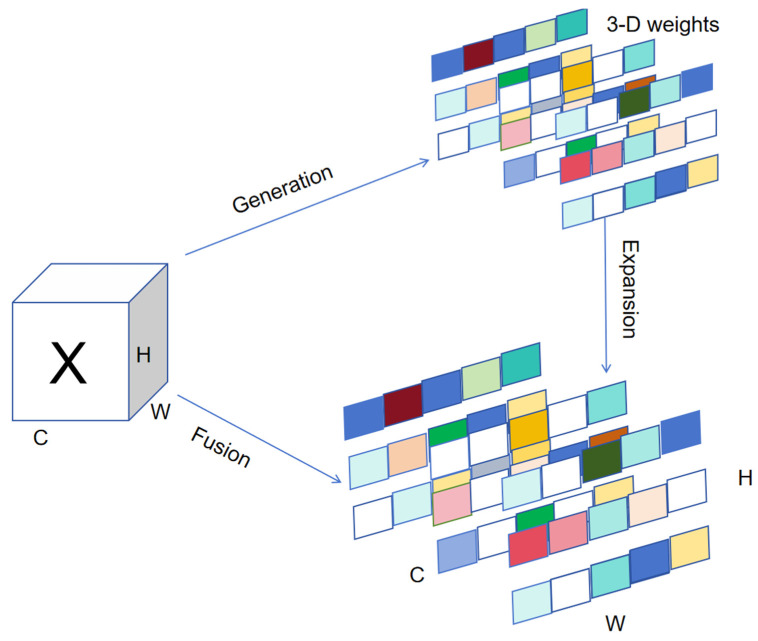
SimAm Module.

After we embedded it into the feature extraction layer of the backbone network, the model was able to adaptively enhance the salient features of pigeon eggs (such as elliptical contours and specific lustre) at zero parameter cost. It allocates weights by deriving analytical solutions through energy functions without introducing any learnable parameters. This not only significantly reduces the risk of model overfitting but also makes it highly suitable for lightweight deployment. Unlike traditional attention mechanisms that only generate one-dimensional channel weights or two-dimensional spatial weights, SimAm assigns a unique three-dimensional weight to each element (C × H × W) of the feature map, achieving fine attention regulation that is coordinated and unified at both the channel and spatial levels. In addition, this module features plug-and-play functionality, enabling seamless integration into existing CNN architectures with extremely low computational overhead, thereby improving performance. At the same time, it suppresses the noise from complex backgrounds, thereby enhancing the ability to perceive tiny targets such as pigeon eggs. The structure of the SimAm module is shown in Figure 4.

#### 2.2.4. WIoU-v3 Loss Function

In the breeding farm, there are three significant problems in the detection of pigeon eggs: First, there is an extreme imbalance in sample quality. Most pigeon eggs in the breeding environment are easy to detect, but the key missed detections occur in a few complex samples. Second, pigeon eggs are tiny, and a deviation of just a few pixels can lead to detection failure. Third, abnormal samples are inevitable in the breeding environment. Pigeon eggs that are partially obscured by feathers or feed, in shaded or strongly reflective environments, or whose shape and colour change in the later stage of incubation, can interfere with model training. However, the traditional IoU loss function and its variants (such as CIoU) assign static gradient gains to all samples, fail to distinguish between simple and complicated samples, and are even less capable of effectively handling Outliers, thereby limiting the further improvement of model performance.

To solve the above problems, this study introduces the third-generation loss function based on WIS-IOU (WIoU-v3) [21]. The core innovation of WIoU-v3 lies in its Dynamic Non-Monotonic Focusing Mechanism. This mechanism can intelligently evaluate the “anomaly degree” of each anchor box and dynamically adjust its gradient gain accordingly, thereby guiding the model to learn more efficiently.

First of all, WIoU-v3 defines an indicator to measure the degree of anomaly of anchor boxes.

(2) $$\beta =\frac{L_{\mathrm{IoU}}}{\bar{L_{\mathrm{IoU}}}}$$

Among them, $L_{\mathrm{IoU}}$ is the IoU loss value of the current anchor box, and $\bar{L_{\mathrm{IoU}}}$ is the Exponential Moving Average (EMA) of $L_{\mathrm{IoU}}$ in the current batch. The larger the β value, the poorer the quality of the anchor box (i.e., the more abnormal it is).

Subsequently, WIoU-v3 constructed a non-monotonic focusing coefficient.

(3) $$r=\frac{\beta }{\delta \alpha^{\beta -\delta }}$$

Among them are hyperparameters. Where δ makes r = 1 when β = δ, and it is applied to WIoU v1 to obtain WIoU v3, that is, LWIoU3 = rLWIoU1. When the degree of outliers of the anchor frame satisfies β = C (where C is a constant), the anchor frame will achieve the highest gradient gain.

Because the quality division standard of the anchor box is dynamic, WIoU-v3 can adapt the gradient gain distribution strategy to suit the current situation at each moment best.

In the pigeon egg detection task, WIoU-v3 can adaptively assign appropriate gradient gains to these complex pigeon egg samples, enhancing the model’s learning of key features while simultaneously avoiding overfitting of a tiny number of abnormal samples. Meanwhile, to reduce the problem of missed detection of pigeon eggs, we adjusted the conf of YOLOv12n-pg to 0.25 and used it in conjunction with the WIoU-v3 loss function.

#### 2.2.5. Data Augmentation and Model Training

**Figure 5:**
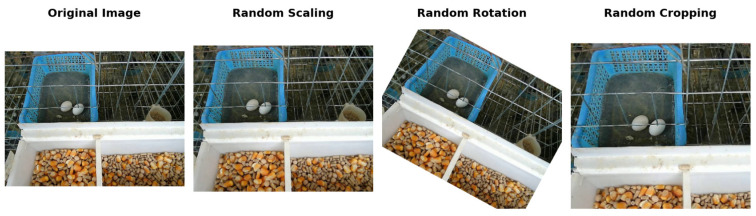
The chart of three data augmentation effects: random cropping, scaling, and rotation.

During the model training process, to expand the diversity of the training dataset, this study adopted geometric transformation methods such as random cropping, scaling, and rotation for Data Augmentation to enhance the model’s generalisation ability and robustness [22]. The effect after data augmentation is shown in Figure 5.

The training parameters of this study are shown in Table 2.

### 2.3. Detection Result Fusion Strategy Based on Time Redundancy and Majority Voting

In the dynamic environment of the breeding farm, the activities of pigeons (such as returning to the nest and turning eggs) and the vibration of the cage caused by the operation of equipment can introduce instantaneous interference, which is manifested explicitly in three typical situations: (a) the brief obstruction of the eggs by the pigeon body (usually less than 3 s), (b) the slight rolling of the position of the pigeon eggs, and (c) the instantaneous blurring of the image. These interferences increase the missed detection rate of single-frame images. To enhance the robustness of the system, this study designs a majority voting fusion mechanism based on time redundancy. The core of this mechanism lies in leveraging the instantaneous nature of interference to increase the probability of occasional correct detection, resulting in a stable system output through sampling in the time dimension.

#### 2.3.1. Time Redundancy and Probability Enhancement

Let the probability of a single frame image successfully detecting pigeon eggs at any moment be $\;P_{d}$. This probability is close to 1 in the ideal state without interference, but it will significantly decrease due to the aforementioned dynamic interference. Assuming the interference event is independent and instantaneous, the probability of its occurrence at a sampling time point is $\;P_{f}$.

This mechanism builds the system’s decision-making on multiple observations by conducting N = 3 independent samplings within one collection cycle. For a real nest site with eggs, the probability that the system ultimately makes a correct judgment of “egg presence” through majority voting (this study sets a threshold T = 2) can be described by a binomial distribution:

(4) $$P_{\mathrm{system}}=\;\sum_{k=T}^{N}\left(\begin{array}{c} N\\ k \end{array}\right){\left(P_{d}\right)}^{k}{\left(1-P_{d}\right)}^{N-k}$$

Among them, $P_{d}$ is equal to 1 minus $P_{f}$. This model indicates that even if single-frame detection fails due to transient interference ($P_{d}$ Less than 1), as long as a sufficient number (≥T) of valid frames can be successfully captured in multiple independent samplings, the probability of the system making a correct judgment as a whole, $P_{\mathrm{system}}$ will be much higher than $P_{d}$. Our sampling strategy (3 random samplings) ensures that the sampling interval (10 s) is longer than the duration of typical interference, thereby satisfying the assumption of event independence and making this probability model valid in practice.

Calculations demonstrate that even if single-frame detection is unreliable due to interference (assuming $P_{d}$ = 0.8), the overall system accuracy. $P_{\mathrm{system}}$ can be increased to 0.896. This model theoretically proves that our temporal redundancy strategy effectively leverages the transient nature of interference to enhance system-level reliability to an industry-acceptable standard through multiple samplings. The sampling interval (10 s) in our system is significantly longer than the duration of typical interference, ensuring the independence of sampling events and validating this probability model in practice.

#### 2.3.2. Fusion Process Implementation

Based on the above theory, we fuse three frames of images collected by the same pigeon nest within one cycle:

Specifically, the camera moves within the same pigeon cage for 30 s. The camera takes an image every ten seconds, and a total of three photos are taken in each pigeon cage. Then, pigeon egg detection is conducted on each image, and the detection results are recorded. Using the majority voting mechanism, the detection results from the three images are combined to determine the final number and location of pigeon eggs.

By counting the number of Negg tests for “egg presence”, when Negg ≥ 2, it is determined as “egg presence”. This method enhances the fault tolerance for transient interference.

### 2.4. Design of Pigeon Egg Positioning Model

This study adopts barcode recognition based on OpenCV to achieve this function. Combined with the image of the pigeon cage scanned by the camera, the position information of the pigeon cage is detected. In conjunction with the pigeon egg target detection technology, the batch output of the “pigeon cage position—pigeon egg quantity” key-value pair is jointly realised.

**Figure 6:**
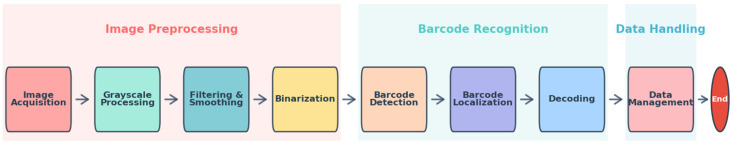
Barcode recognition method flowchart.

Hang barcode signs in the pigeon cages and use OpenCV to scan and recognise the barcodes in the camera footage. Match the barcode scanning results with the number of eggs checked. The specific process is shown in Figure 6.

#### 2.4.1. Barcode Design

The barcode adopted in this research is the EAN-13 standard barcode. Firstly, the quiet zone design on both sides of the barcode provides a precise positioning reference for the image processing algorithm and effectively solves the interference problem caused by the grid background in the breeding environment. Secondly, barcodes have relatively distinct rectangular features [23], with a fixed aspect ratio (approximately 2:1). In contrast, similar-shaped ones, such as pigeon cage grids, pigeon nests, and drinking cups, do not have a 2:1 ratio. Therefore, most non-target contours can be excluded in the preprocessing stage, significantly improving the recognition efficiency.

In actual pigeon farms, there are problems such as cage contamination, egg liquid splashing causing barcode contamination, and barcode displacement due to cage vibration. We have made special optimisations in the material selection and manufacturing process of the barcodes. We have chosen a PET base material with a thickness of 0.2 mm and a matte lamination process. The choice is mainly based on the excellent physical and chemical properties of PET materials. First, it features extremely high mechanical strength and dimensional stability. It is less likely to deform or wrinkle due to fluctuations in temperature and humidity in the breeding farm, ensuring the constancy of the barcode’s geometric characteristics. Secondly, the PET material itself has excellent waterproof, moisture-proof, and chemical corrosion resistance properties. It can effectively resist the corrosion caused by water vapour, faeces, and ammonia in the pigeon loft environment, preventing identification failure due to material degradation. This ensures the mechanical strength of the barcode and effectively suppresses the recognition interference caused by the reflection of ambient light.

The number of digits of the barcode required for this study is 8. The first and second digits represent the number of pigeon houses, with a value range of 1 to 25. The 3rd to 4th digits represent the number of pigeon cages in the same building, with a value range of 1 to 4. The number of pigeon cages in the same group from the 5th to the 6th positions, with a value range of 1 to 99. The 7th to 8th digits are the numbers of the pigeon nests in the same cage, with a value range of 1 to 6. Thus, the barcode design work of this study is completed.

#### 2.4.2. Barcode Extraction

**Figure 7:**
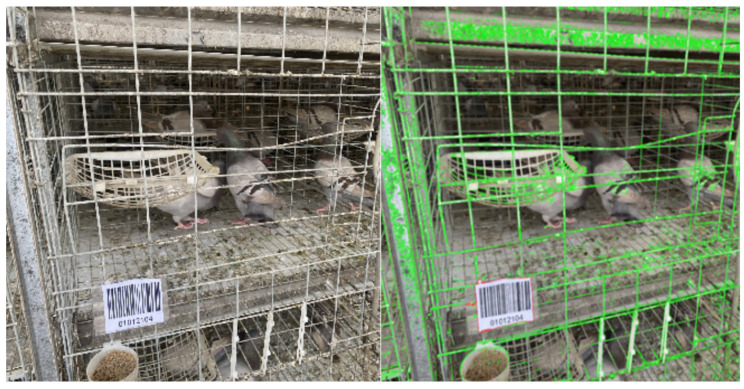
Comparison of barcode images before and after contour detection. The right image shows the identified contours (green dots) and the successfully located barcode boundary (red bounding box) after processing.

Since the image captured by the camera contains the pigeon cage and the barcode, it is necessary to extract the barcode separately to facilitate subsequent processing and decoding. Although the pigeon cages, drinking cups and some breeds of pigeons are all white, which poses a challenge to the recognition of barcodes, this study successfully achieved accurate recognition and distinction of barcodes by comprehensively considering the white and rectangular features of barcodes and designing the shooting position and Angle of the camera to reduce background interference. Therefore, in this study, the image is converted into grayscale, processed through filtering and smoothing, and then binarised using a threshold. Combined with the contour detection technology [24], the contours in the image are found and represented by green dots. By calculating the area of each contour, the largest rectangular contour was identified and marked with a red dot, representing the boundary of the barcode. By obtaining the bounding box coordinates of the largest rectangular contour, the position and size of the barcode in the image can be determined, as shown in Figure 7.

#### 2.4.3. Grayscale Processing

In this study, the barcode images captured by the camera were in RGB format. To reduce the computational complexity of subsequent processing and focus on the luminance information of the image, the input image is first grayscaled. This process essentially involves mapping the three-dimensional RGB color space to a one-dimensional grayscale space. This study employs the weighted average method, which is widely used in digital image processing. The theoretical basis for this is that the human eye has different sensitivities to light of different wavelengths, with green light being the most sensitive, followed by red light, and blue light being the weakest. The functional relationship between RGB values and grey level is shown in Equation (1) below.

(5) Y=R∗0.229+G∗0.587+B∗0.114

Among them, R, G, and B, respectively, represent the intensities of the red, green, and blue components of the pixels in the original image. This coefficient set (commonly known as the ITU-R BT.601 standard [25]) can generate grayscale images that conform to the perceptual characteristics of the human eye, providing high-quality input for subsequent image analysis.

#### 2.4.4. Filtering and Smoothing Processing

During the process of image acquisition and transmission, images are inevitably contaminated by impulse noise (such as salt-and-pepper noise) and random noise [26,27]. To suppress noise while retaining the edge structure information of the barcode to the greatest extent—which is crucial for the subsequent contour extraction—this study selects median filtering as the preprocessing method [28].

Median filtering is a nonlinear signal processing technique. Its core principle is to scan the image with a sliding window (or convolution kernel), sort the gray values of all pixels within the window, and replace the original value of the central pixel of the window with the median. This operation can effectively filter out isolated noise points and provide excellent protection for step edges (such as the bar and space boundaries of barcodes), avoiding the edge blurring problem that may be caused by linear filters (such as mean filtering).

The key to the filtering effect lies in the size selection of the sliding window. A window that is too large will overly smooth the image, resulting in the loss of details. A window that is too small may not effectively filter out noise. Through the analysis of the image noise characteristics in the breeding farm environment, this study ultimately selected a 3 × 3 pixel square window as the sampling template, achieving the best balance between denoising effect and detail retention.

#### 2.4.5. Binarisation Processing

The requirements for barcode processing in this study are high speed, low time complexity and sound effect. Therefore, the noise processing algorithm adopted in this study is the OTSU algorithm (Maximum Between-Class Variance Algorithm).

The OTSU algorithm is based on the least squares method and is an adaptive threshold determination method. Its characteristics are good applicability and a relatively simple algorithm [29]. Inter-class variance refers to the degree of difference between the foreground and the background, with the formula being:

(6) $$\sigma \_b^{2}(t)=w_{0}\left(t\right)*w_{1}\left(t\right)*{\left[\mu_{0}\left(t\right)-\mu_{1}\left(t\right)\right]}^{2}$$

Here, $w_{0}\left(t\right)$ and $w_{1}\left(t\right)$ respectively represent the proportions of the pixels on both sides of the threshold t, $\mu_{0}\left(t\right)$ and $\mu_{1}\left(t\right)$ respectively represent the average grey values of the pixels on both sides of the threshold t.

The algorithm traverses all possible thresholds t (from 0 to 255), calculates the corresponding inter-class variance $\sigma \_b^{2}(t)$, and selects the t that maximizes $\sigma \_b^{2}(t)$ as the final binarization threshold. This method does not require manual threshold setting and can adaptively respond to changes in the overall brightness and contrast of the image, ensuring the robustness of barcode extraction.

#### 2.4.6. Contour Detection and Extraction

To precisely locate the barcode region from the preprocessed image, a contour-based detection approach was employed. The image was first processed using the Canny edge detection algorithm to accentuate the rectangular boundaries of the barcode. The Canny algorithm was selected for its optimal performance in balancing noise robustness and edge localization, achieved through a multi-stage process: (1) smoothing the image with a Gaussian filter to suppress noise, (2) computing gradient magnitude and direction, (3) applying non-maximum suppression to thin edges, and (4) utilizing double thresholding with hysteresis to finalize strong edges and connect weak ones.

Subsequently, all potential contours in the image were extracted through the cv2.findContours function in the OpenCV library. To accurately identify barcodes, these contours were strictly screened, and those that did not conform to barcode characteristics, such as having too small an area or abnormal aspect ratios, were excluded.

Specifically, an area threshold was set to eliminate overly small noise profiles, and the boundary rectangles of each profile were calculated. A 2:1 aspect ratio was used as the key indicator to determine whether it was a barcode. This study also utilised the cv2.minAreaRect function to obtain the minimum bounding rectangle of the contour. Further, it analysed the direction and size of the contour to ensure that the recognised contour conforms to the geometric features of the barcode.

#### 2.4.7. The Positioning Function of Pigeon Eggs

The implementation steps of pigeon egg positioning in this study mainly fall into the following three points:

(1) Use OpenCV’s cv2.barcode_BarcodeDetector to decode barcode information and extract valid information;
(2) Call the egg-checking model algorithm to obtain the number of eggs;
(3) Send the position of the pigeon eggs (8 bits) and the quantity of pigeon eggs to the database in the form of key-value pairs.

The barcode feature extraction, achieved by combining OpenCV to obtain the coding result, provides data support for the precise positioning of pigeon eggs in the subsequent process. Based on the above, the key-value pairs of the positioning information and quantity information of the pigeon eggs are obtained and stored in the database.

### 2.5. Integration and Deployment of the Visual Management System

**Figure 8:**
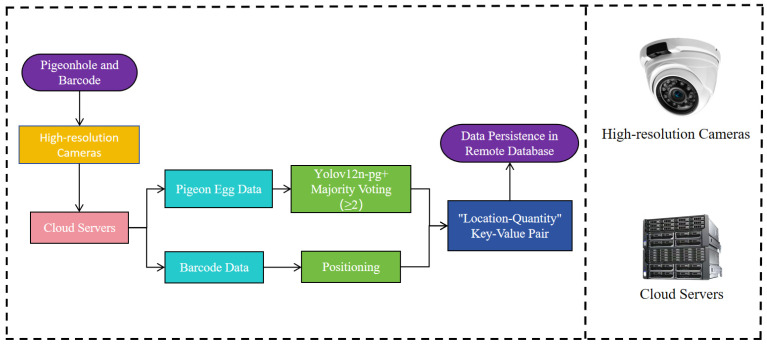
Core workflow of the vision-based intelligent management system.

To ensure the engineering reproducibility and practical applicability of the proposed system, this section details the deployment scheme and operational workflow of the vision-based intelligent management system. The system was architected to form a closed-loop pipeline, encompassing hardware setup, software integration, and automated data management. Its core workflow is shown in Figure 8.

The hardware configuration was centered around a Hikvision T12HV3-IA/PoE industrial camera, chosen for its robustness and suitability for the agricultural environment. This camera was mounted on a custom-designed bracket attached to the feeding machine, positioning it at a lateral top-down angle of approximately 30° and a consistent distance of 50 cm from the pigeon cages. This precise positioning was critical to simultaneously capture clear, unobstructed views of both the eggs within the nest and the EAN-13 barcode affixed to the exterior of the cage, all within a single frame. The system’s operation was contingent on a stable lighting environment. In cases of insufficient natural light within the pigeon loft, soft artificial supplementary lighting was provided to ensure consistent image quality without causing stress to the pigeons.

At the software level, the system operated on a framework built upon PyTorch for the deep learning inference and OpenCV for traditional image processing tasks. The automated workflow, designed to integrate seamlessly with the feeding routine, proceeded as follows: the movement of the feeder served as the primary trigger. Upon arriving at a specific pigeon cage, the system initiated a data acquisition cycle during the feeder’s brief pause. Over this 30 s window, the camera captured three images at precise 10 s intervals, a strategy explicitly designed to create temporal redundancy against transient disturbances. Subsequently, these three frames were processed in parallel: one stream was fed into the YOLOv12n-pg model for egg detection, while the other was processed by the OpenCV-based barcode recognition algorithm. The egg detection results from the three frames were then fused using the pre-defined majority voting mechanism, where a cage was confirmed to contain eggs if at least two out of the three frames registered a positive detection. This final egg count was automatically associated with the positional data decoded from the barcode. Ultimately, this “position-quantity” key-value pair was transmitted and written in real-time to a remote database, thereby completing a full management closed-loop from physical observation to digital record-keeping.

## 3. Results and Analysis

### 3.1. Model Training Results and Analysis

After 300 cumulative iterations of training on the deep learning host, the dataset of this study obtained a model for pigeon egg object detection. The training results are shown in Table 3.

As shown in Table 3, the YOLOv12n-pg model proposed in this study achieves the best balance in comprehensive performance. In the comparison with other lightweight models and RT-DETR-L, the advantages of this model are particularly obvious. Its mAP50-95 reaches 83.6%, which is the highest level among the listed lightweight models but also significantly better than the 68.1% of RT-DETR-L [30], demonstrating its superior comprehensive detection accuracy.

More importantly, this model has extremely prominent advantages in terms of computational efficiency and model complexity: the computational load is reduced to 4.9 GFLOPs, which is significantly lower than that of similar lightweight models, and is only 4.7% of the computational load of RT-DETR-L. The parameter quantity is only 1.56 M, which is reduced by approximately 31.3% to 39.5% compared to other lightweight models, and is less than 5% of the parameter quantity of RT-DETR-L at the same time. The final model size was compressed to 3.5 MB.

To further verify the superiority of the YOLOv12n-pg model, this study systematically compared it with the larger-scale YOLOv12s and YOLOv12m models in the YOLOv12 series. As shown in Table 3, YOLOv12s and YOLOv12m, as medium-scale models in this series, perform exceptionally well in detection accuracy, with mAP50-95 reaching 83.5% and 83.1%, respectively, slightly higher than the basic YOLOv12n model.

However, this slight improvement in accuracy comes at the cost of a significantly increased model complexity. The computational load of YOLOv12s reaches 19.3 GFLOPs, with a parameter count of 9.07 M and a model size of 18.7 MB. The computational load of YOLOv12m is as high as 58.6 GFLOPs, with 19.54 M of parameters and a model size of 39.6 MB. Compared with these medium-sized models, YOLOv12n-pg maintains a higher detection accuracy (83.6% for mAP50-95), while the computational complexity is only 25.4% of YOLOv12s and 8.4% of YOLOv12m, respectively. The number of parameters was only 17.2% of that of YOLOv12s and 8.0% of that of YOLOv12m, respectively.

**Figure 9:**
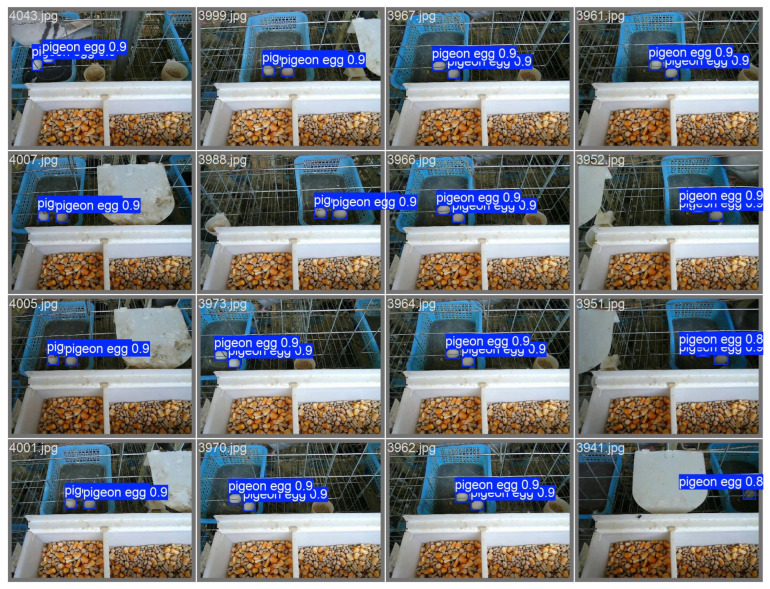
The test results of pigeon eggs detection.

These indicators fully demonstrate that through structural optimisation, this model has achieved a slight improvement in accuracy while significantly reducing computing and storage overhead, showcasing higher hardware deployment efficiency and practicality. Meanwhile, in the test set, the improved model significantly solved the problem of missed detections, as shown in Figure 9.

### 3.2. Module Validity Verification

This study verified the effectiveness of the proposed improved strategy through modular ablation experiments. The specific data comparison is shown in Table 4:

The improvement module of the detection head mainly contributes to the lightweighting and efficiency enhancement of the model. After introducing this module separately, the number of model parameters was significantly reduced to 1.55 M, the computational load dropped to 4.5 GFLOPs, and the model size was compressed to 3.4 MB. This indicates that the designed small detection head effectively reduces the model complexity, and the mAP50-95 index remains the same as the baseline, suggesting that this module maintains the basic performance of the model while significantly reducing parameters.

The SimAm attention module focuses on enhancing feature representation capabilities. After introducing this module separately, both the Recall rate and mAP50 saw slight improvements, indicating that it improved the model’s perception and recognition ability of the target. However, this module brings particular computational overhead, with the computational load increasing to 6.3 GFLOPs, the number of parameters slightly higher than the baseline, and a slight decrease in mAP50-95, indicating that it may introduce redundant computations.

The introduction of the WIoU-v3 loss function further optimises the training process of the model. As shown in the last row of Table 4, after adopting the WIoU-v3 loss function, the model’s mAP50-95 on the test set reached 83.6%, which was 0.8 percentage points higher than that of the baseline model. Meanwhile, the recall rate significantly increased to 99.6%, effectively solving the problem of missed detection of pigeon eggs. It is worth noting that WIoU-v3 has achieved performance improvement without adding any parameters or computational costs. This is attributed to its dynamic non-monotonic focusing mechanism, which effectively enhances the regression effect on complex samples and optimises the prediction accuracy of bounding boxes.

Most crucially, by introducing the WIoU-v3 loss function and adjusting conf to 0.25, the model achieved a significant improvement in Recall (from 98.1% to 99.6%). Although its Precision has slightly declined (from 99.4% to 98.1%), this performance trade-off is fully in line with the core design goals of this study. In the business scenario of automated management of pigeon eggs, the core value of the system lies in minimising the missed inspection of genuine pigeon eggs to the greatest extent. Missed inspections will directly lead to the absence of asset statistics, distorted reproduction records and economic losses. In contrast, a small number of false detections can be effectively filtered and corrected through the system-integrated multi-frame majority voting mechanism. Therefore, in system design, prioritising the guarantee of an extremely high recall rate is a crucial engineering decision.

**Figure 10:**
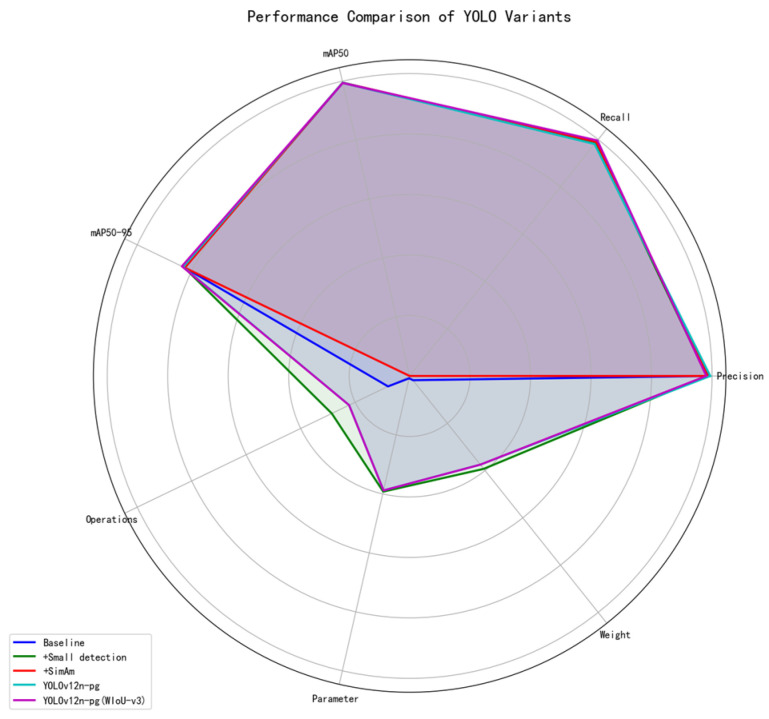
Radar chart comparing the performance of different model variants from the ablation study. All metrics (Precision, Recall, mAP50, mAP50-95) are normalized such that larger values are better, while computational metrics (Operations, Parameters, Weight) are reverse-normalized for consistent interpretation. The chart shows the balanced performance of the final YOLOv12n-pg model.

As shown in Figure 10, the final fusion scheme YOLOv12n-pg effectively combines the advantages of the three modules and achieves the best balance between accuracy and efficiency. The experimental results show that the final model, while maintaining high Precision (Precision: 98.1%, mAP50: 99.4%) and high Recall rate (Recall: 99.6%), has seen the MAP50-95 increase to 83.6%, significantly outperforming the baseline model. Furthermore, the model’s parameter quantity is only 1.56 M, and the computational cost is 4.9 GFLOPs. This achieves a good balance between accuracy and efficiency, verifying the effectiveness and practicability of the proposed improvement strategy.

### 3.3. Model Generalisation Test

To verify the generalisation of the model, this study analysed the efficacy of the proposed YOLOv12n-pg (WIoU-v3) model’s practical application in different pigeon farms. The test data originated from a pigeon farm scene that was completely independent of the training environment. We randomly selected 16 images for in-depth testing.

**Figure 11:**
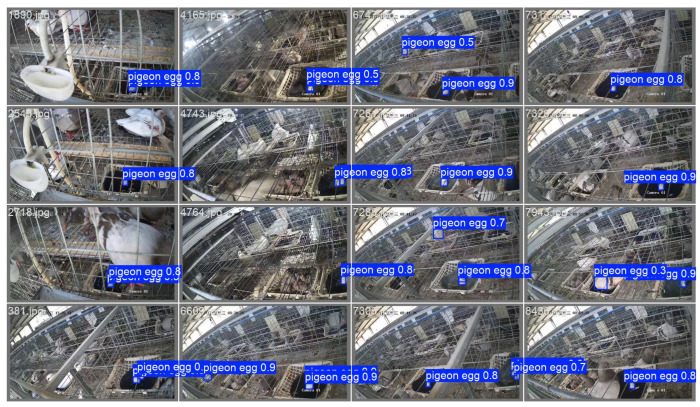
The results of the pigeon eggs detection in the new pigeon farm.

The test results show that the model demonstrates outstanding generalisation ability in unknown scenarios. The accuracy rate reaches 95.0%, proving that the model has excellent anti-interference ability and can effectively distinguish pigeon egg targets from complex backgrounds. The recall rate is 96.2%, indicating that the model has an extremely high recognition coverage rate for the target pigeon eggs, and the phenomenon of missed detections has been effectively controlled. The mAP50 index remained at a high level of 94.2%, fully verifying the model’s stable detection performance in unfamiliar environments, as shown in Figure 11.

From a practical application perspective, the high recall rate is of great significance for monitoring breeding production, ensuring comprehensive management of pigeon egg assets. The high accuracy rate ensures the reliability of the system output and effectively avoids the waste of resources caused by false alarms. It is particularly worth noting that in test scenarios with significant differences in environmental conditions, the core detection indicators of the model only show minor fluctuations compared to the performance of the training set. This phenomenon strongly confirms the effectiveness of the improvement strategy proposed in this paper. The lightweight detection head design significantly reduces computational complexity while maintaining the model’s performance, and the SimAm attention mechanism enhances the model’s environmental adaptability by strengthening the discriminative nature of features. The WIoU-v3 loss function enhances the model’s processing ability for differentiated samples through a dynamic optimisation mechanism.

The results of this study verified the applicability and stability of the proposed method in various pigeon farm environments from a practical perspective. This provides a reliable technical solution for deploying the automated pigeon egg detection system, positively impacting the development of the innovative breeding industry.

### 3.4. Model Improvement Visualisation

**Figure 12:**
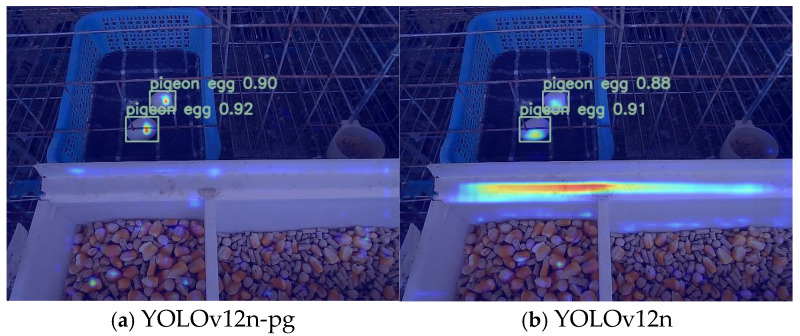
Comparison of feature visualization heatmaps generated by Grad-CAM. The color map encodes feature importance, with warmer hues (e.g., red, yellow) highlighting regions of high importance and cooler hues (e.g., blue, green) corresponding to areas of lesser importance. (**a**) The improved YOLOv12n-pg model shows highly concentrated activation strictly on the pigeon egg. (**b**) The baseline YOLOv12n model exhibits dispersed attention, including irrelevant background areas (e.g., the white trough), indicating vulnerability to interference.

In this study, the Grad-CAM [31] (gradient-weighted class Activation Mapping) technique was adopted to conduct feature visualisation analysis on the original YOLOv12n model and the improved YOLOv12n-PG model. Through the generated heatmap, we can visually observe the degree of attention the model pays to different image regions during the reasoning process, thereby deeply understanding the decision-making mechanism of the model. The red areas in the heatmap represent the parts that the model pays high attention to, while the blue areas indicate the parts that receive less attention. Under the same experimental scenario of pigeon egg detection, the two models exhibit significantly different feature focusing patterns. The heatmap distribution of the original YOLOv12n model shows that, in addition to the normal focus on the pigeon egg itself, the model has an abnormally high activation response to the white background area of the trough. This phenomenon of excessive focus on the background indicates that the model is vulnerable to environmental interference factors, which may lead to unstable detection performance. In contrast, the heatmap of the improved YOLOv12n-pg model shows a highly concentrated feature selection pattern. The thermal activation area is strictly limited within the contour range of pigeon eggs and has almost no significant response to the white background of the trough. This precise feature focusing ability proves that the improved model can effectively distinguish the target object from the environmental background, avoiding unnecessary distraction of attention, as shown in Figure 12.

### 3.5. Model Test Results and Analysis

To test the accuracy of the pigeon egg target detection model and the effect of the majority voting mechanism, 200 pigeon eggs were randomly placed in the pigeon houses of breeding pigeons that had not laid eggs. The system operation at 6:00, 14:00 and 18:00 was tested. The testing process lasted for seven consecutive days, with tests conducted at the three fixed times mentioned above every day. The test results are shown in Table 5.

From the model checking results, it is evident that in each period, the missed detection rate of the model is less than or equal to 1.5%. After the majority voting mechanism was adopted, the false detection rate further decreased, effectively achieving precise identification of pigeon eggs. Meanwhile, the 30 s processing time for a single pigeon position also meets the efficiency requirements. Therefore, from the perspective of the core functions of “counting” and “positioning”, the accuracy and efficiency of the current model are sufficient to support its initial deployment in pigeon farms.

### 3.6. Analysis of the Mitigation Effect of the Multi-Frame Voting Mechanism on Dynamic Interference

**Figure 13:**
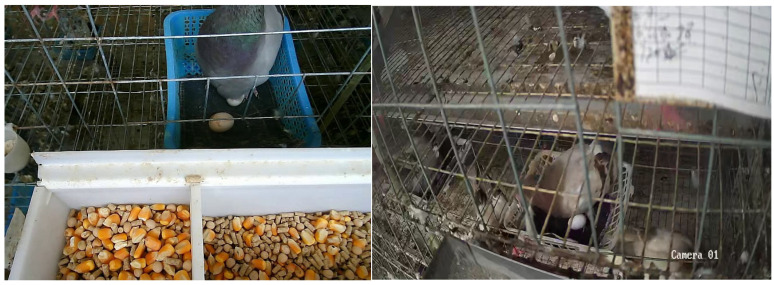
An example of a missed detection scenario that the multi-frame voting mechanism could not resolve, where pigeon eggs were persistently occluded by the parent pigeon across multiple sampling frames, highlighting the boundary condition of the temporal redundancy strategy.

To quantitatively evaluate the mitigation effect of the multi-frame voting mechanism on dynamic interference in the aquaculture site, this study conducted a retrospective analysis of the causes of all missed samples in the test set. The results show that among the total of 35 missed samples, only four can be clearly attributed to the continuous occlusion of pigeons (i.e., pigeons did not leave their nests at more than two sampling time points). In comparison, the remaining 31 missed samples were caused by other factors (such as extreme lighting, background interference, etc.). This data powerfully demonstrates that the multi-frame voting mechanism has an outstanding ability to alleviate typical instantaneous dynamic interference, as shown in Figure 13.

#### 3.6.1. Mitigation of Temporary Occlusion and Slight Positional Changes

The results show that the number of missed samples directly caused by slight positional changes in eggs due to pigeons’ brief stays (<3 s) or activities is 0. This proves that the multi-frame voting mechanism successfully filters out these transient interferences. The working principle is that the brief occlusion of pigeons or the slight rolling of eggs are low-probability events over time and are unlikely to occur multiple times in more than half of the sampled frames. Therefore, when most frame capture pigeon eggs are unobstructed, the mechanism can output the correct “egg presence” judgment, thereby eliminating potential instantaneous missed detection risks at the system level.

#### 3.6.2. Robustness Against Image Blurring

This study did not identify any missed samples that could be solely attributed to image blurring caused by cage vibration. This indirectly indicates that the degradation of image quality caused by vibration is usually transient, and its degree has not yet exceeded the feature extraction ability of the YOLOv12n-pg model. The role of the multi-frame voting mechanism in this process is to reduce further the impact of single-frame image quality fluctuations on the final result. As long as sufficiently clear images can be obtained at most sampling moments, slight blurring of individual frames will not affect the final decision, thus ensuring the system’s stability in a slightly vibrating environment.

#### 3.6.3. The Performance Boundary and Discussion of the Mechanism

Although the multi-frame voting mechanism performs well against the above-mentioned transient interference, there is a clear boundary to its performance. Figure 10 clearly shows its working mechanism and boundaries: For brief interference, most clear frames can correct a few disturbed frames; however, for persistent occlusion, most frames fail, causing the mechanism to malfunction. All four missed detections caused by occlusion in this study belong to this kind of “persistent occlusion” boundary situation. This, from the opposite perspective, confirms that the effectiveness of the multi-frame voting mechanism is highly dependent on one premise: dynamic interference is sparse in time rather than continuous. Our sampling strategy successfully covered the spatiotemporal characteristics of most of the disturbances at the breeding site. However, for the long-term hatching behaviour of pigeons, other perception methods (such as thermal imaging) need to be introduced for collaborative judgment.

### 3.7. The Sufficiency of the Dataset Size

We demonstrated the sufficiency of the dataset from two dimensions: model performance and application requirements. In terms of performance dimensions, our model achieved outstanding results with a mAP50 of 99.4% and a recall rate of 99.6% on independent test sets. The core metrics have entered the saturation range, and the marginal benefits brought by further increasing the data are limited. In terms of application dimensions, the ultimate goal of the system is to precisely count the number of pigeon eggs and correlate their locations. The test results show that after the majority voting mechanism, the system’s missed detection rate has been stabilised within 1%, fully meeting the core industrial requirements of breeding farms for the accuracy of asset statistics. Therefore, whether in terms of the learning level of the model or the efficiency in solving practical problems, the scale of the current dataset is sufficient and efficient.

### 3.8. Location Results and Analysis of Pigeon Eggs

**Figure 14:**
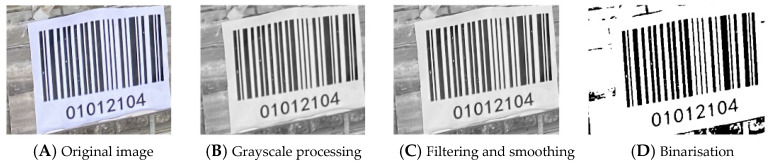
Effects after barcode preprocessing.

The barcode image processing result is shown in the following Figure 14:

The number and location information of the obtained pigeon eggs were uploaded to the database, as shown in the following table. The information on the pigeon eggs was smoothly transmitted to the database, making it easily accessible to the management personnel, as shown in Table 6.

## 4. Discussion

### 4.1. Balance Analysis of the Accuracy and Efficiency of Pigeon Egg Positioning

In the automated pigeon egg management system, there is a contradiction between positioning accuracy and processing efficiency, and the optimal balance point needs to be sought based on the actual application scenarios. The multi-frame voting mechanism reflects a typical trade-off between accuracy and efficiency. By conducting three samplings on the same pigeon nest, the system reduced the missed detection rate to 0.5%, but the total detection time increased to three times that of the single-frame mode. This design is suitable for the scenario of slow-moving feeders, but a more optimised solution is needed on high-efficiency production lines.

The weight of precision and efficiency requirements varies in different application scenarios: The performance monitoring of breeding pigeons can accept a relatively low efficiency to ensure data accuracy [32]. Daily production management needs to take both into account. Mass production, on the other hand, places more emphasis on efficiency [33]. This system ultimately controls the processing time of a single cage position within 30 s, which not only matches the working rhythm of the feeder but also ensures practical positioning accuracy. This balance is dynamic. In the future, through an adaptive computing resource allocation mechanism, more refined adjustments can be achieved based on the complexity of the scenarios.

### 4.2. Discussion on the Applicability of Various Variants of Poultry Eggs

In the practical application of poultry egg detection, the system needs to deal with the morphological differences in different poultry egg varieties. Although this study used pigeon eggs as the detection object for algorithm optimisation, its technical framework has the potential to handle various variants of poultry eggs. As a typical small poultry egg, the successful detection of pigeon eggs verified the algorithm’s ability to capture small-scale targets, which means that the system can be extended to detect smaller-sized eggs, such as quail eggs.

However, there are significant differences in morphological characteristics among different poultry eggs. Larger poultry eggs, such as chicken eggs and duck eggs, have more significant curvature changes and size fluctuations, which require the detection algorithm to have stronger scale invariance [34,35,36]. The current model structure, based on a single detection head, may need to be adjusted in such scenarios by introducing moderate multi-scale feature fusion to ensure detection stability. Meanwhile, the surface texture features of different poultry eggs also vary. For instance, duck eggs often have bluish-white patches on their surfaces, while quail eggs have special spot patterns. These feature changes require the algorithm to have a stronger generalisation ability in the feature extraction stage [37,38].

Future system expansion can be achieved through transfer learning strategies, which fine-tune the pre-trained pigeon egg detection model using specific poultry egg data. This method can not only maintain the efficiency advantage of the original algorithm, but also quickly adapt to new detection tasks [39,40]. At the same time, considering the differences in demand in various production scenarios, the algorithm should provide configurable parameter interfaces, allowing users to adjust the detection sensitivity according to the specific characteristics of poultry eggs, achieving a balance between universality and specialisation.

### 4.3. System Limitations and Future Directions

Two aspects need improvement in this study: The issue of missed detection of pigeon eggs persists due to long-term occlusion by pigeons and dim lighting. In the future, multimodal fusion technology can be explored [41]. Secondly, the automatic identification of the biological status of pigeon eggs (fresh eggs, fertilised eggs, rotten eggs) will be a key extension to enhance the practical value of the system [42,43,44], which requires the introduction of new sensing technologies such as multispectral imaging [45,46].

### 4.4. Financial Cost and Commercialisation Potential Analysis

The technical path selection of the system constructed in this study itself contains a deep consideration of economic feasibility. The financial cost advantage of the system is rooted in the lightweight design of its core algorithm. Through targeted model pruning and optimisation, the system can run stably on low-cost embedded hardware, which fundamentally avoids the huge expenses brought by deploying high-performance computing devices. This “algorithm-driven cost optimisation” strategy provides a prerequisite for the large-scale popularisation of technology in resource-constrained agricultural production environments.

In terms of commercial potential, the value proposition of the system lies in transforming the traditional labour-intensive quality inspection process into a highly automated data collection process. Its economic benefits are not only reflected in the direct substitution effect of labour costs, but also in the fact that, through precise “location-quantity” data assets, it provides the possibility for the refined management and scientific decision-making of breeding farms, thereby creating incremental value beyond cost reduction and efficiency improvement. This value leap from “replacing human labour” to “empowering management” is the key to distinguishing it from traditional automated equipment and building a core business barrier [47,48].

Regarding the expected scale, the modular and lightweight architecture of the system endows it with a high degree of scalability. From a technical perspective, the marginal cost growth of system deployment is nearly linear, which means it can maintain a good cost–benefit ratio in farms of different scales (from small and medium-sized farmers to large groups). Meanwhile, to promote the inclusiveness of technology, it is suggested to adopt differentiated discount schemes: provide basic discounts to professional cooperatives and family farms, and offer additional benefits to farmers included in the industrial assistance system.

## 5. Conclusions

This study successfully addressed the long-standing challenges in the pigeon egg breeding industry—namely, inefficient manual inspection, high egg breakage rates, and coarse management practices—by designing and constructing an intelligent pigeon egg detection and positioning system. This system integrates a novel lightweight object detection model with robust visual positioning technology to establish a comprehensive automated management framework.

The principal conclusion drawn from this research is that a holistic system, combining a purpose-built, lightweight deep learning model, a dynamic anti-interference strategy, and automated data association, can effectively automate the core tasks of pigeon egg management. The developed YOLOv12n-pg model demonstrates that through targeted architectural optimisation, it is feasible to achieve an optimal balance between high detection accuracy and operational efficiency.

Furthermore, the study concludes that temporal redundancy strategies, such as the implemented multi-frame voting mechanism, offer a robust and practical solution for mitigating the transient dynamic interference prevalent in real-world breeding environments. This approach significantly enhances system-level reliability beyond what is achievable with single-frame detection.

Finally, the integration of visual egg detection with barcode-based positioning conclusively enables a paradigm shift from traditional, experience-dependent management to a data-driven approach. The automated generation of “location-quantity” key-value pairs lays the foundation for refined farming practices, enabling deeper operational analysis and more informed decision-making.

In summary, this work not only presents a technically efficient and economically feasible solution for automated pigeon egg management but also provides a reusable methodological framework and an engineering paradigm for the visual inspection of small targets in dynamic and complex agricultural settings.

## Figures and Tables

**Table 1:** Deep learning host parameters.

Name	Parameters
CPU	12th Gen Intel(R) Core(TM) i9-12900H (Intel, Santa Clara, CA, USA)
GPU	RTX 3060
CUDA	12.1
TensorFlow	2.4.1
Python	3.12.11
PyTorch	2.5.1

**Table 2:** Pigeon egg object detection training parameters.

Parameters	Values
Epochs	300
Class	1
Batch-size	16
Image-size	640 × 640 × 3
Learning rate 0	0.01
Weight_decay	0.0005
Optimizer	SGD

**Table 3:** Model detection results.

Module	Precision/%	Recall/%	mAP50/%	mAP50-95/%	Operations/GFLOPS	Parameter/10^6^	Weight/MB
YOLOv10n	97.1	98.8	99.4	83.0	6.5	2.27	5.8
YOLOv11n	98.6	98.8	99.4	82.7	6.3	2.58	5.5
YOLOv12n	98.8	98.6	99.4	82.8	5.8	2.53	5.5
YOLOv13n	98.9	98.5	99.4	82.3	6.2	2.45	5.4
YOLOv12s	98.1	99.5	99.5	83.5	19.3	9.07	18.7
YOLOv12m	98.0	99.5	99.4	83.1	58.6	19.54	39.6
RT-DETR-L	96.3	91.7	94.7	68.1	103.4	31.99	66.2
YOLOv12n-pg (WIoU-v3)	98.1	99.6	99.4	83.6	4.9	1.56	3.5

**Table 4:** Ablation Experiment Results (Test Set Performance).

Module	Precision/%	Recall/%	mAP50/%	mAP50-95/%	Operations/GFLOPS	Parameter/10^6^	Weight/MB
Baseline	98.8	98.6	99.4	82.8	5.8	2.53	5.5
Small detection	98.3	98.7	99.5	82.8	4.5	1.55	3.4
SimAm	98.6	99.0	99.5	82.5	6.3	2.55	5.6
YOLOv12n-pg	99.4	98.1	99.5	83.1	4.9	1.56	3.5
YOLOv12n-pg(WIoU-v3)	98.1	99.6	99.4	83.6	4.9	1.56	3.5

Note: The second and third lines indicate that only this module is used. The last line demonstrates that YOLOv12n-pg adopts the WIOU-v3 loss function.

**Table 5:** Model detection results.

Time	Whether to Adopt a Majority Vote	Daily Sample Number	Average Number of Missed Detections	Missed Detection Rate
6:00	No	200	2	1
	Yes	200	1	0.5
14:00	No	200	1	0.5
	Yes	200	1	0.5
18:00	No	200	3	1.5
	Yes	200	1	0.5

**Table 6:** Database table.

Id	Uid	Number	Time
1	01012104	1	06-28 15:33:57
2	01012105	1	06-28 15:34:27
3	01012106	2	06-28 15:34:55
4	01022104	1	06-28 15:35:29
5	01022106	1	06-28 15:35:56
6	01032306	0	06-28 15:36:28

## Data Availability

The data presented in this study are available on request from the corresponding author.
